# *Pereskia aculeata* Muller (Cactaceae) Leaves: Chemical Composition and Biological Activities

**DOI:** 10.3390/ijms17091478

**Published:** 2016-09-03

**Authors:** Lucèia Fàtima Souza, Lucia Caputo, Ingrid Bergman Inchausti De Barros, Florinda Fratianni, Filomena Nazzaro, Vincenzo De Feo

**Affiliations:** 1 Department of Pharmacy, University of Salerno, 84084 Fisciano (Salerno), Italy; luceia.souza@ufrgs.br (L.F.S.); lcaputo@unisa.it (L.C.); 2 Department of Agronomy, University of Rio Grande do Sul (UFRGS), 91501-970 Porto Alegre, Brazil; ingridb@ufrgs.br; 3 Istituto di Scienze dell’Alimentazione, Consiglio Nazionale delle Ricerche (ISA-CNR), 83100 Avellino, Italy; florinda.fratianni@isa.cnr.it (F.F.); mena@isa.cnr.it (F.N.)

**Keywords:** *Pereskia aculeata*, essential oil, leaf extracts, antioxidant activity, antibacterial activity, antifungal activity, cytotoxicity, adenylate cyclase (ADCY)

## Abstract

The aims of this work were to study the chemical composition of the essential oil from the leaves of *Pereskia aculeata* and to evaluate some biological activities of three leaf extracts. The phenolic content, antioxidant activity, and in vitro antimicrobial and antifungal activities were determined. The methanol extract showed antioxidant activity (EC_50_ 7.09 mg/mL) and high polyphenols content (15.04 ± 0.31 mg gallic acid equivalents (GAE)/g). The petroleum ether extract exhibited potent antibacterial activity against *Escherichia coli*, whereas the chloroform extract showed inhibitory activity against *Bacillus cereus* and *Staphylococcus aureus*. The petroleum ether and methanol extracts were more effective in inhibiting the growth of *Aspergillus versicolor*. The possible cytotoxicity of extracts on neuroblastoma SH-SY5Y cancer cell line and the influence on adenylate cyclase (ADCY) expression was also studied. *P. aculeata* chloroform extract showed antiproliferative activity with an IC_50_ value of 262.83 µg/mL. Treatments of SH-SY5Y neuroblastoma cells with 100 µg/mL of methanol extract significantly reduced ADCY1 expression.

## 1. Introduction

The genus *Pereskia* is considered the least advanced from Cactaceae family, possessing succulent leaves and terminal flowers gathered in cymes. These plants are generally climbing species native to South America, adapted to low altitudes and naturally distributed from south to northeast of Brazil [1,2].

The plant is popularly known as “ora pro nobis” and is used in cooking because of its high nutritional content. The leaves of *P. aculeata* contain high levels of proteins when compared to other plants commonly used for human food [3,4] and they also contain high levels of minerals, dietary fiber, vitamins A and C, and folic acid [5].

In folk medicine, the leaves of *P. aculeata* are used as emollients, due to their high mucilaginous content, in skin wound healing, and to treat inflammation [1,2].

Only a little information is available in the literature about the biological activities of *P. aculeata.* Pinto and coworkers [6] reported that some extracts of *P. aculeata* inhibited the breast cancer cell line MCF-7 and proliferation human promyelocytic leukemia cells HL60 cell, and that phenolic compounds are the major antioxidant components in *P. aculeata* leaves. Sitosterol, stigmasterol, flavonoids, and phenols are reported to be in the leaves of *P. aculeata* [1].

Some *Pereskia* species are reported to be used as natural remedies for headache, inflammation, gastric pain, for pain relief, and as tonics [7]. *P. bleo* DC. and *P. grandifolia* hort. ex Peiff. showed anti-inflammatory, antioxidant, antifungal, antimicrobial and cytotoxic activities [1,7,8].

In this work, we studied the chemical composition of the essential oil from leaves of *P. aculeata* and the polyphenol composition of three leaf extracts. Moreover, the extracts have been evaluated for their in vitro antioxidant, antibacterial and antifungal activities. Additionally, their cytotoxicity and the possible effects on central nervous system have been studied.

## 2. Results

### 2.1. Essential Oil Yield and Composition

Hydrodistillation of the aerial part of *P. aculeata* yielded 0.02% (on a dry mass basis) of a pale yellow essential oil. Table 1 shows the chemical composition of the essential oil; compounds are listed according to their elution order on an HP-5 MS column. In all, 24 compounds were identified, accounting for 91.03% of the total oil. Oxygenated sesquiterpenes were the main constituents of the oil (44.92%), the main compound being acorone (30.0%). Other important compounds are (*Z*,*Z*)-methyl-4,6-hexadecadiene (16.34%), 1-nonadecen-ol (6.18%) and (5*E*,9*E*)-farnesyl acetone (5.70%).

### 2.2. Free Radical-Scavenging Capacity

**Figure 1:**
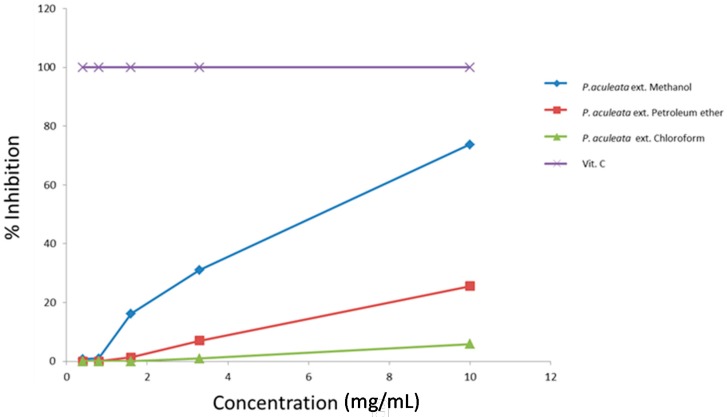
Antioxidant activity (percent of inhibition) of *Pereskia aculeata* extracts.

The antioxidant activity of *P. aculeata* extracts was assessed by 2,2-diphenyl-1-picrylhydrazyl (DPPH) assay, evaluating the H-donating or radical-scavenging ability of the oils using the stable radical 2,2-diphenyl-1-picrylhydrazyl (DPPH) as a reagent. Figure 1 shows the antioxidant activity of the three extracts of *P. aculeata*. Methanolic extract shows the highest percentage of inhibition of DPPH (73.71%) at a concentration of 10 mg/mL. The EC_50_ values demonstrate that methanol and petroleum ether extracts showed high antioxidant activity (EC_50_ values of 7.09 and 18.27 mg/mL, respectively). The chloroform extract exhibited low DPPH scavenging activity, with an EC_50_ value of the 81.09 mg/mL (Table 2). Ascorbic acid was used as a standard antioxidant.

### 2.3. Total Phenolic Compounds

The total polyphenols content in the three extracts is presented in Table 3. Methanol and petroleum ether extracts showed the highest amounts of polyphenols (15.04 ± 0.67 and 11.78 ± 0.23 mg gallic acid equivalents (GAE)/g, respectively). The chloroform extract contains 5.17 ± 0.41 mg GAE/g.

### 2.4. Antimicrobial and Antifungal Activity

In Table 4 and Table 5 we report the widths (mm) of the inhibition halos of the three extracts tested at 1–4 μg, using different bacteria and molds. The petroleum ether extract inhibited the growth of all tested strains and exhibited potent antibacterial activity against *Escherichia coli*. The chloroform extract showed inhibitory activity against the Gram-positive pathogens, *Bacillus cereus* DSM 4313, *B.* *cereus* DSM 4384, and *Staphylococcus aureus*. The chloroform and methanol extracts did not have significant activity against *Escherichia coli*. The antimicrobial activity appears to be dose-dependent. The methanol extract was inactive at 1 µg/mL against *Bacillus cereus* DSM 4313 and *Staphylococcus aureus*, but at a concentration of 4 µg/mL, the inhibition of these strains was superior to tetracycline against the same microorganisms.

A difference in antifungal activity of the three extracts against four fungal strains was observed: *Penicillium expansum*, *P. citrinum*, and *Aspergillus niger* were not susceptible to the concentration of the 1 and 2 µg/mL for petroleum ether and chloroform extracts, whereas petroleum ether and methanol extracts were more effective in inhibiting the growth of *Aspergillus versicolor.*

### 2.5. Cytotoxicity of Pereskia aculeata Extracts

**Figure 2:**
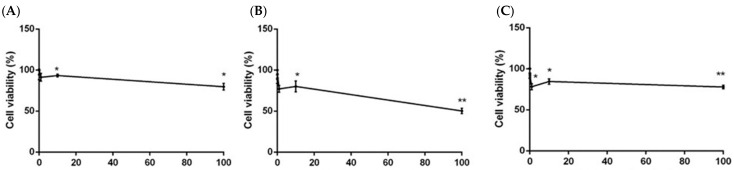
Cell viability calculated as percentage after MTT assay. Cells were treated with different concentrations (0.01–100 µg/mL) of petroleum ether (**A**), chloroform (**B**), and methanol (**C**) extracts of *P. aculeata*, for 24 h and solvent (DMSO, 0.1%) alone. Data are the mean ± SD of three experiments * *p* < 0.05, ** *p* < 0.01, vs. DMSO.

The treatment of SH-SY5Y neuroblastoma cells with 0.01–100 µg/mL of the three extracts for 24 h resulted in a low cytotoxic activity. Petroleum ether and methanol extracts showed an IC_50_ > 2000 µg/mL and IC_50_ > 1600 µg/mL, respectively. However, the treatment with chloroform extract resulted in a stronger cytotoxicity (IC_50_ < 300 µg/mL) (Figure 2).

### 2.6. Adenylate Cyclase 1 (ADCY1): Western Blot Analysis

**Figure 3:**
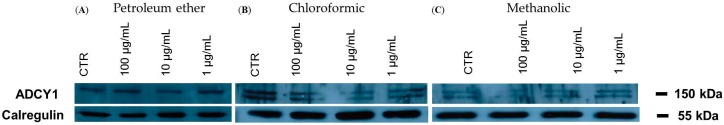
Representative Western blot of adenylate cyclase 1 (ADCY1) protein in SH-SY5Y cells treated with petroleum ether (**A**); chloroform (**B**); and methanol (**C**) extracts of *P. aculeata*.

**Figure 4:**
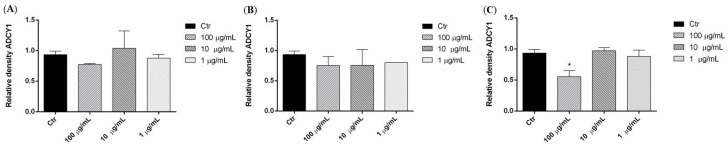
Relative expression levels of the ADCY1 protein in SH-SY5Y cells treated with petroleum ether (**A**), chloroform (**B**), methanol (**C**) extracts. Each panel shows the densitometric analysis of bands in the treated groups and control. Values are the mean ± SD in each group (*n* = 3). * *p* < 0.05, compared to control (ANOVA followed by Dunnett’s multiple comparison test).

We investigated the effects of three *P. aculeata* extracts in SH-SY5Y human neuroblastoma cells. Representative Western blots and quantitative densitometric analysis for adenylate cyclase 1 (ADCY1) protein expression in SH-SY5Y following exposure to different concentrations of the extracts are shown in Figure 3. Treatments of SH-SY5Y neuroblastoma cells with 100 µg/mL of methanol extract of *P. aculeata* for 24 h significantly reduced ADCY1 expression (Figure 4C), however, petroleum ether and chloroform extracts showed no effects on ADCY1 expression (Figure 4A,B).

## 3. Discussion

Very few reports are available in the literature about the essential oils obtained both from *Pereskia* species and other Cactaceae. In a previous research, Souza and coworkers [9] reported the chemical composition of the essential oil obtained from the leaves of *P. aculeata.* In this oil, the main compounds were phytol (29.4%), hexadecanoid acid (17.4%), and linoleic acid (12.7%). Our data showed that in *P. aculeata* essential oil, these compounds are present in minor amounts, with differences in the relative proportion of the constituents. These variations may be attributed mainly to environmental conditions, age of the plant, method of harvesting, and method used to isolate the essential oil [10].

The stronger scavenging activity showed by methanol and petroleum ether extracts than in the chloroform extract was probably due to the concentration of antioxidant compounds. Our data disagree from those reported in literature about antioxidant activities of *Pereskia* species. Recently, Pinto and coworkers reported the antioxidant activity of different fractions from the methanol extract of *P. aculeata* by DPPH bioautographic analysis [6]. An interesting activity was observed in the hexane fraction due to the presence of phenolic compounds. Wahab and coworkers reported a hexane extract as the most powerful antioxidant if compared to ethyl acetate, dichloromethane, and methanol extracts [11]. Sim and coworkers [7] found a hexane extract of *P. bleo* with lowest EC_50_ value (210 µg/mL) among different extracts in DPPH assay; the same authors reported an ethyl acetate extract of *P. grandifolia* as the best DPPH-scavenging activity, followed by hexane and methanol extracts [12].

The results of Table 4 show that there is a correlation between higher DPPH-scavenging activity and the content of total phenolics. This agrees with previous reports showing that phenolic compounds generally correlate with antioxidant activities measured by DPPH assay [7,13]. Our results suggest that *P. aculeata* can be considered as a source of polyphenols compared to other plants such as *Hypericum perforatum* that showed a percentage of total polyphenols of the 2.12 mg GAE/g for methanolic extract and 0.79 mg GAE/g for chloroform extract [14]. In literature, little is known about the phenolic content of species of *Pereskia*: Sim and coworkers found that ethyl acetate extracts of *P. bleo* and *P. grandifolia* possessed high total phenolic content (40.12 and 45.99 mg GAEs/g, respectively) [7,12].

Different types of microorganisms were chosen to investigate the antimicrobial activity using the inhibition halo technique. This test is often used to assess antibacterial activity of vegetal extracts and essential oils [15,16]. Nevertheless, several limitations should be noted, such as lack of standardization of inoculum density, adequate culture medium, agar viscosity, and size and number of specimens per plate. Other disadvantages are that this method is relatively insensitive and semiquantitative and does not distinguish between bacteriostatic or bactericidal properties of the substances tested. In addition, the results of this test do not depend on only the toxicity of the material for the particular microorganism, but are also influenced by the diffusibility of the material across the medium. A material that diffuses more easily will probably provide larger zones of inhibition [17]. However, each susceptibility test has inherent advantages and limitations and agar-based methods like E-test and agar disk diffusion represent valid methods compared, for example, to the broth microdilution method [18].

The antimicrobial activity exhibited by *P. aculeata* extracts against both Gram-positive (*B. cereus* and *S. aureus*) and Gram-negative (*E. coli* and *P. aeruginosa*) bacteria may indicate the presence of a broad spectrum of compounds with antibiotic activity. The crude extracts of plants tend to contain a mixture of molecules that vary in chemical structure and composition, which in turn may influence the biological actions [19]. In literature, the antimicrobial activities of crude extracts of some *Pereskia* species are reported only for crude extracts of some *Pereskia* species. Philip and coworkers evaluated the antimicrobial activity of different extracts of *P. bleo* and *P. Haw* [20]. An ethyl acetate extract of *P. grandifolia* showed antimicrobial activity against *P. aeruginosa*, *S. aureus*, and *B. subtilis*; methanol and ethyl acetate extracts of *P. bleo* were effective against *P. aeruginosa*, and the ethyl acetate extract was also active against *B. subtilis.* In our study, the methanol extract from *P. aculeata* demonstrated strong antibacterial activity against *P. aeruginosa*. These results are comparable with those reported by Wahab and coworkers, which showed high and moderate activity against *P. aeruginosa* and *Salmonella choleraesuis*, exerted by hexane and methanol extracts from *P. bleo* [11].

The extracts from *P. aculeata* showed different antifungal activity (Table 5). Petroleum ether and methanol extracts were effective against *Aspergillus versicolor*, producing halos ranging from 2.33 to 9.33 mm and 2.33 to 6.66 mm, respectively. On the other hand, the first extract, at a concentration of 4 µg/mL, was effective against *P. citrinum* and *A. versicolor*. The presence of sterols, such as sitosterol [8,11,21], probably determined the ability of such extracts to inhibit the antifungal activity. Phytosterols are reported for their antibacterial and antifungal activities [22].

The cytotoxic activity of the extracts from *P. aculeata* were evaluated in human neuroblastoma cell line (SH-SY5Y). The IC_50_ values of the tested extracts were >200 µg/mL, indicating that the extract was not cytotoxic, as judged by the criterion set by the National Cancer Institute which stated that the extracts with IC_50_ < 20 µg/mL were considered cytotoxic against the treated cells [23]. Instead, hexane, dichloromethane and ethyl acetate fractions of a *P. aculeata* extract showed cytotoxicity against MCF-7 cells and HL60 cells [6]. Moreover, *P. bleo* methanol extract has been reported for its cytotoxicity on T47-D breast carcinoma cells [24] and a methanol extract of *P. grandifolia* exhibited cytotoxicity against human SAOS-2 osteosarcoma cells [25].

Many plant species are used as sedatives, hypnotics, tranquilizers, treatments for disorders of the central nervous system (CNS) [26]. Previous studies have shown that some extracts of cactaceous species can affect the CNS. Kim and coworkers [27] revealed that the expression levels of brain-derived neurotrophic factor, phosphorylated cyclic AMP (cAMP) response element binding protein, and phosphorylated extracellular signal-regulated kinase (pERK) were significantly increased in hippocampal tissue after 7 days of the administration of a methanol extract of *O. ficus-indica* var. *saboten* administration. Extracts and isolated compounds from *P. bleo* leaf showed antinociceptive activity [28,29]; *P. bleo* fractions had the isolated sitosterol and vitexin which possessed a central antinociceptive effect [29]. Part of this effect is mediated by opioid receptors and the nitrergic pathway. Adenylyl cyclase is involved in the production of the second messenger cyclic AMP (cAMP) in response to various stimuli such as synaptic plasticity, learning, and memory [30,31]. In this perspective, we carried out experiments to determine whether exposure to methanol, petroleum ether and chloroform extracts of *P. aculeata* can affect this pathway in SH-SY5Y cells. Our results showed that high concentration of methanol extracts (100 µg/mL) inhibited ADCY1 expression in SH-SY5Y cell and consequently the intracellular production of cAMP. Clinical and epidemiologic research suggest that reduction of ADCY1 expression has well-documented benefits, including benefits for heart disease and pain [32].

## 4. Materials and Methods

### 4.1. Plant Materials

Leaves of *Pereskia aculeata* were collected in the campus of the Universidade Federal do Rio Grande do Sul (Porto Alegre, Brazil) in October 2015. The plant was identified by Mara Rejane Ritter. A voucher specimen (ICN 155346) was deposited at the Herbarium of the Botanic at the Universidade Federal do Rio Grande do Sul.

### 4.2. Extraction Procedure

One kilogram of leaves were air-dried and then extracted, at room temperature, successively with solvents of increasing polarity (petroleum ether, chloroform, and methanol). Finally, each extract was evaporated to dryness under reduced pressure. The extracts yield was 1.5%, 0.9%, and 2.1% for petroleum ether, chloroform and methanol, respectively.

### 4.3. Isolation of the Volatile Oil

One hundred grams of dried leaves of *P. aculeata* was ground in a Waring blender and then subjected to hydrodistillation for 3 h according to the standard procedure described in the *European Pharmacopoeia* (2004) [33]. The oil was solubilized in *n*-hexane, filtered over anhydrous sodium sulphate and stored under N_2_ at +4 °C in the dark, until tested and analyzed.

### 4.4. GC-FID Analysis

Analytical gas chromatography (GC) was carried out on a Perkin-Elmer sigma-115 gas chromatograph (Pelkin-Elmer, Waltham, MA, USA) equipped with a flame ionization detector (FID) and a data handling processor. The separation was achieved using a HP-5 MS fused-silica capillary column (30 m × 0.25 mm i.d., 0.25 µm film thickness) (Agilent, Roma, Italy). Column temperature: 40 °C, with 5 min initial hold, and then to 270 °C at 2 °C/min, 270 °C (20 min); injection mode splitless (1 µL of a 1:1000 *n*-hexane solution). Injector and detector temperatures were 250 °C and 290 °C, respectively. Analysis was also run by using a fused silica HP Innowax polyethylenglycol capillary column (50 m × 0.20 mm i.d., 0.25 µm film thickness) (Agilent). In both cases, helium was used as carrier gas (1.0 mL/min).

### 4.5. GC/MS Analysis

Analysis was performed on an Agilent 6850 Ser. II apparatus (Agilent), fitted with a fused silica DB-5 capillary column (30 m × 0.25 mm i.d., 0.33 µm film thickness) (Agilent), coupled to an Agilent Mass Selective Detector MSD 5973 (Agilent); ionization energy voltage 70 eV; electron multiplier voltage energy 2000 V.

Mass spectra were scanned in the range 40–500 amu, scan time 5 scans/s. Gas chromatographic conditions were as reported in the previous paragraph; transfer line temperature, 295 °C.

### 4.6. Identification of the Essential Oil Components

Most constituents were identified by gas chromatography by comparison of their Kovats retention indices (Ri) (determined relative to the *t*_R_ of *n*-alkanes (C10–C35)), with either those of the literature [34,35,36,37] and mass spectra or both columns with those of authentic compounds available in our laboratories by means NIST 02 and Wiley 275 libraries [38]. The components’ relative concentrations were obtained by peak area normalization. No response factors were calculated.

### 4.7. Free Radical-Scavenging Capacity

#### 4.7.1. Sample Preparations

Aliquots of three extracts were solubilized in methanol to obtain a final concentration ranging from 10 to 0.4 mg/mL.

#### 4.7.2. DPPH Radical

The methodology based on sequestering the 2,2-diphenyl-1-picrylhydrazyl (DPPH) radical was used to determine the antioxidant activity of the extracts [39]. A 0.1 mL aliquot of each dilution of the extract was reacted with 3.9 mL of DPPH radical (0.06 mM). The readings were made in a spectrophotometer Thermo scientific Multiskan GO at 515 nm after 45 min. For preparation of the standard curve, different concentrations of DPPH methanol solutions (0–60 µM) were used. The DPPH concentration (EC_50_ of µM) in the reaction medium was calculated from the following calibration curve, determined by linear regression: (*R*^2^: 0.9937).

The scavenging capability of test extracts was calculated using the following Equation (1):

DPPH scavenging activity (%) = 100 × [(absorbance control−absorbance sample)/absorbance control](1)

Ascorbic acid (5 µg/mL) was used as reference drug. The experiments were performed in triplicate and averaged.

### 4.8. Total Phenolic Compounds

The total phenolic content was determined using the Folin–Ciocalteu method, described by Singleton and Rossi (1965) [40]. Eight hundred microliters of deionized water and 50 µL of the Folin–Ciocalteu reagent were added to 50 µL of the suitably diluted extract. The mixture was kept for 6 min, then 100 µL of a 7% aqueous Na_2_CO_3_ solution was added. After 120 min, the absorption was measured at 760 nm against water as a blank, using a Cary UV–Vis spectrophotometer (Varian, Palo Alto, CA, USA). The amount of total phenolics was expressed as mg gallic acid equivalents (GAE)/g of extract.

### 4.9. Antimicrobial Assays

The antibacterial activity was evaluated in vitro, by means of the test of the inhibition halo on the plate [16]. The activity of *P. aculeata* extracts was tested on five species of bacteria: *Staphylococcus aureus* (DMS 25923), *Bacillus cereus* (DSM 4313), and *Bacillus cereus* (DSM 4384), representative of the Gram-positives; *Escherichia coli* (DMS 857) and *Pseudomonas aeruginosa* (ATCC 50071) for Gram-negatives. Bacteria were purchased from DSMZ, Braunschweig, Germany. Microbial strains were previously grown in Nutrient Broth (Sigma, Milano, Italy), at 37 °C for 18 h. The microbial suspensions (1 × 10^7^ colony-forming units (CFU)/mL) were uniformly distributed on nutrient agar plates in sterile conditions. Different amounts of extracts (1, 2, and 4 µg/mL) were spotted on the inoculated plates. After 10 min, plates were incubated at 37 °C for 24–48 h depending on the strain, under sterile conditions. The antimicrobial activity was evaluated by measuring the diameter (in mm) of the zone of inhibition. A disk treated with DMSO alone served as the negative control, tetracycline (7 µg/disc; Sigma Aldrich Italy, Milano, Italy) was used as a positive control. The experiments were performed in triplicate and averaged.

### 4.10. Antifungal Activity

Some fungal strains of agro-food interest, *Penicillium citrinum* (DSM 1997), *P. expansum* (DSM 1994) and *Aspergillus versicolor* (DSM1943), were used for antifungal activity. The strains were purchased from DSMZ. Sterile Whatman No.1 paper filter disks (Ø = 5 mm) were individually placed on the inoculated plates (Ø = 90 mm dishes) and impregnated with different amounts of the extracts (1, 2, and 4 µg/mL, corresponding to 4–16 µL), were used. A cell suspension of fungi was prepared in sterile distilled water, adjusted to contain 10^6^ CFU/mL, and 50 µL were plated onto potato dextrose agar (PDA) (Sigma Aldrich Italy). After 20 min under sterile conditions at room temperature, plates were incubated at 28 °C until the mycelium of fungi reached the edges of the control plate (negative control without the extracts) [16]. The resulting clear zones of inhibition were measured in mm. DMSO (10 µL) was used as a negative control. Samples were tested in triplicate and the results are expressed as mean ± standard deviation.

### 4.11. Cell Cultures

Human neuroblastoma (SH-SY5Y) cancer cells were cultured in Roswell Park Memorial Institute Medium (RPMI) supplemented with 1% l-glutamine, 10% heat-inactivated fetal bovine serum (FBS), 1% penicillin/streptomycin (all from Sigma Aldrich, St. Louis, MO, USA) at 37 °C in an atmosphere of 95% O_2_ and 5% CO_2_.

### 4.12. MTT Assay

Cells were plated (5 × 10^3^) in 96-well culture plates in 150 µL of culture medium and incubated at 37 °C in humidified 5% CO_2_. The day after, a 150 µL aliquot of serial dilutions of the three extracts (0.01–100 µg/mL) was added to the cells and incubated for 24 h. DMSO alone was used as control. Cell viability was assessed through MTT (3-(4,5-dimethylthiazol-2-yl)-2,5-diphenyl tetrazolium bromide) assay. Briefly, 30 µL of MTT (5 mg/mL) was added and the cells incubated for additional 3 h. Thereafter, cells were lysed and the dark blue crystals solubilized with 30 µL of a solution containing 50%, *v*/*v*, *N*,*N*-dimethylformamide, 20%, *w*/*v*, SDS with an adjusted pH of 4.5. The optical density (OD) of each well was measured with a microplate spectrophotometer (Thermo Scientific Multiskan GO) equipped with a 520 nm filter. Cell viability in response to treatment was calculated as a percentage of control cells treated with DMSO at the final concentration 0.1% viable cells = (100 × OD treated cells)/OD control cells [41].

### 4.13. Extraction Proteins and Western Blotting

Cells were treated with different concentrations (0.01–100 µg/mL) of the three extracts and, after 24 h, were collected and lysed using Laemmli buffer to extract total proteins. For Western blot analysis, an aliquot of total protein was run on 8% SDS-PAGE gels and transferred to nitrocellulose. Nitrocellulose blots were blocked with 10% nonfat dry milk in Tris buffer saline 0.1% Tween-20 over night at 4 °C and incubated with primary anti-ADCY1 (Santa Cruz Biotechnology, Santa Cruz, CA, USA) for 3 h at room temperature. Immunoreactivity was detected by sequential incubation with horseradish peroxidase-conjugated secondary antibody (Amersham Biosciences, Pittsburgh, PA, USA) and enhanced chemiluminescence reagents (ImmunoCruz, Santa Cruz Biotechnology) [42].

### 4.14. Statistical Analysis

All experiments were carried out in triplicate. Data of each experiment were statistically analyzed using GraphPad Prism 6.0 software (GraphPad Software Inc., San Diego, CA, USA) followed by comparison of means (two-way ANOVA) using Dunnett’s multiple comparisons test, at the significance level of *p* < 0.05.

## Figures and Tables

**Table 1:** Essential oil composition of *Pereskia aculeata*.

No.	Compound	%	Ki ^a^	Ki ^b^	Identification ^c^
1	(*E*)-β-Ionone	0.75	1488	1476	1,2
2	Dihydro-β-agarofuran	0.57	1519	1503	1,2
3	*cis*-Dihydro-mayurone	0.17	1551	1595	1,2
4	Caryophyllene oxide	0.51	1575	1583	1,2
5	α-Muurolol	0.22	1643	1642	1,2,3
6	*ar*-Tumerone	1.10	1654	1668	1,3
7	14-Hydroxy-(*Z*)-caryophyllene	0.29	1661	1667	1,2
8	(*Z*)-3-Hexenyl salicylate	0.17	1664	1669	1,2
9	14-Hydroxy-9-*epi*-(*E*)-caryophyllene	0.28	1668	1669	1,2
10	2-hexyl-(*E*)-cinnamaldehyde	0.60	1734	1749	1,2
11	1-Octadecene	0.62	1783	1790	1,2,3
12	2-Ethylhexyl salicylate	1.73	1789	1807	1,2
13	Acorone	30.0	1824	1820	1,2
14	Cyclopentadecanolide	5.48	1830	1833	1,2
15	1-Nonadecen-ol	6.18	1848		1,2
16	(*Z*,*Z*)-Methyl-4,6-hexadecadiene	16.34	1866		1,2
18	(5*E*,9*E*)-Farnesyl acetone	5.70	1903	1913	1,2
19	Methyl hexadecanoate	4.92	1910	1921	1,2,3
20	Isopropyl hexadecanoate	0.42	1984	2024	1,2
21	Methyl linoleate	4.44	2075	2085	1,2
22	Methyl octadecanoate	0.69	2107	2125	1,2,3
23	Linoleic acid	4.74	2126	2133	1,2
24	Phytol	5.11	2148		1,2
	**Total compounds**	91.03			
	Oxygenated monoterpenes	15.96			
	Monoterpene hydrocarbons	0.62			
	Oxygenated sesquiterpenes	44.92			
	Non-terpene	24.42			
	Diterpene hydrocarbons	5.11			

^a^ Kovats retention index on HP-5 MS column; ^b^ Kovats retention index on HP Innowax; ^c^ 1 = Kovats retention index, 2 = mass spectrum, 3 = coinjection with authentic compound.

**Table 2:** Straight equation used to calculate EC_50_ antioxidant activity of *Pereskia aculeata* extracts.

Sample	Equation	*R*^2^	EC_50_
Petroleum ether	*y* = 0.3072*x* − 6.1561	0.9811	18.27 mg/mL
Chloroform	*y* = 0.6346*x* − 1.0909	0.8999	81.09 mg/mL
Methanol	*y* = 7.8743*x* − 5.8489	0.9938	7.09 mg/mL

Antioxidant activity is expressed as the EC_50_ mg/mL of 2,2-diphenyl-1-picrylhydrazyl (DPPH).

**Table 3:** Content of polyphenols in their extracts of *Pereskia aculeata.*

	Ether Extract mg GAE/g	Chloroform Extract mg GAE/g	Methanol Extract mg GAE/g
Total polyphenols	11.78 ± 0.23	5.17 ± 0.41	15.04 ± 0.67

The data are mean values and standard deviations.

**Table 4:** Antibacterial activity of petroleum ether, chloroform and methanol extracts of *P. aculeata.*

Extract	*B. cereus* 4313	*B. cereus* 4384	*S. aureus*	*P. aeruginosa*	*E. coli* 8579
Petroleum ether					
1 µg/mL	7.60 ± 0.47 ***	3.66 ± 0.47 ****	13.00 ± 1.41 ****	4.0± 00 ****	10.66 ± 0.47 ****
2 µg/mL	11.33 ± 0.94	5.66 ± 0.47 ****	16.66 ± 1.24 ****	7.33 ± 0.94 ***	18.33 ± 1.59 ****
4 µg/mL	14.66 ± 0.47 ****	10.0 ± 00	20.33 ± 00 ****	10.66 ± 1.24	20.66 ± 0.94 ****
Chloroform					
1 µg/mL	2.00 ± 00 ****	7.66 ± 0.94 ****	5.66 ± 0.47	8.66 ± 0.47 ***	0
2 µg/mL	4.33 ± 0.47 ****	9.33 ± 0.47 *	8.33 ± 1.24 ****	8.66 ± 0.47 ***	0
4 µg/mL	6.33 ± 0.47 ****	9.33 ± 0.47 *	10.33 ± 0.47 ****	10.33 ± 0.47	0
Methanol					
1 µg/mL	0	7.66 ± 0.47 ****	0	6.33 ± 0.47 ****	0
2 µg/mL	5.33 ± 0.47 ****	8.66 ± 0.47 **	7.6 ± 1.88 *	10.66 ± 0.47	0
4 µg/mL	10.66 ± 00	8.66 ± 0.47 **	8.33 ± 1.24 ***	13.33 ± 1.24 ****	0
DMSO					
Control	10.5 ± 0.5	10.5 ± 0.5	6.0 ± 0.5	10.5 ± 0.5	6.0 ± 0.5

Dunnett’s test vs. Tetracycline 7 µg. **** *p* < 0.001; *** *p* < 0.01; ** *p* < 0.1; * *p* < 0.5.

**Table 5:** Antifungal activity of petroleum ether, chloroform, and methanol extracts of *P. aculeata.*

Extract	*P. expansum*	*P. citrinum*	*A. niger*	*A. versicolor*
Petroleum ether				
1 µg/mL	–	–	–	2.33 ± 0.47
2 µg/mL	–	–	–	6.66 ± 0.47
4 µg/mL	5.33 ± 0.47	5.33 ± 0.47	2.00 ± 00	9.33 ± 0.47
Chloroform				
1 µg/mL	–	–	–	–
2 µg/mL	–	–	–	–
4 µg/mL	–	2.66 ± 0.47	–	5.00 ± 00
Methanol				
1 µg/mL	–	–	–	2.33 ± 0.47
2 µg/mL	2.70 ± 0.47	–	–	5.00 ± 00
4 µg/mL	5.33 ± 0.47	4.66 ± 00	–	6.66 ± 00

## References

[1] Sharif K.M., Rahman M.M., Zaidul I.S.M., Jannatul A., Akanda M.J.H., Mohamed A., Shamsudin S.H. (2013). Pharmacological relevance of primitive leafy Cactuses *Pereskia*. Res. J. Biotechnol..

[2] Pinto N.D.C.C., Scio E. (2014). The biological activities and chemical composition of *Pereskia* species (Cactaceae)—A review. Plant Food Hum. Nutr..

[3] Almeida M.E.F., Corrêa A.D. (2012). Utilization of cacti of the genus *Pereskia* in the human diet in a municipality of Minas Gerais. Ciência Rural.

[4] Martinevski C.S., Oliveira V.R., Rios A.D.O., Flores S.H., Venzke J.G. (2013). Utilization of Bertalha (*Anredera cordifolia* (Ten.) Steenis) and ora pro nobis (*Pereskia aculeata* Mill.) in preparing breads. Braz. J. Food Nutr..

[5] Takeiti C.Y., Antonio G.C., Motta E.M., Collares-Queiroz F.P., Park K.J. (2009). Nutritive evaluation of non-conventional leafy vegetable (*Pereskia aculeata* Miller). Int. J. Food Sci. Nutr..

[6] Pinto N.C.C., Santos R.C., Machado D.C., Florêncio J.R., Fagundes E.M.Z., Antinarelli L.M.R., Scio E. (2012). Cytotoxic and antioxidant activity of *Pereskia aculeata* Miller. Pharmacologyonline.

[7] Sim K.S., Sri Nurestri A.M., Sinniah S.K., Kim K.H., Norhanom A.W. (2010). Acute oral toxicity of *Pereskia bleo* and *Pereskia grandifolia* in mice. Pharmacogn. Mag..

[8] Malek S.N.A., Shin S.K., Wahab N.A., Yaacob H. (2009). Cytotoxic components of *Pereskia bleo* (Kunth) DC. (Cactaceae) leaves. Molecules.

[9] Souza L.F., de Barros I.B., Mancini E., de Martino L., Scandolera E., de Feo V. (2014). Chemical composition and biological activities of the essential oils from two *Pereskia* species grown in Brazil. Nat. Prod. Commun..

[10] Misharina T.A. (2001). Influence of the duration and conditions of storage on the composition of the essential oil from coriander seeds. Appl. Biochem. Microbiol..

[11] Wahab S.I.A., Abdul A.B., Mohan S.M., Al-Zubairi A.S., Elhassan M.M., Ibrahim M.Y. (2009). Biological activities of *Pereskia*
*bleo* extracts. Int. J. Pharmacol..

[12] Sim K.S., Sri Nurestri A.M., Norhanom A.W. (2010). Phenolic content and antioxidant activity of *Pereskia grandifolia* Haw. (Cactaceae) extracts. Pharmacogn. Mag..

[13] Tabart J., Kevers C., Pincemail J., Defraigne J.O., Dommes J. (2009). Comparative antioxidant capacities of phenolic compounds measured by various tests. Food Chem..

[14] Del Monte D., de Martino L., Marandino A., Fratianni F., Nazzaro F., de Feo V. (2015). Phenolic content, antimicrobial and antioxidant activities of *Hypericum perfoliatum* L.. Ind. Crops Prod..

[15] Nazzaro F., Fratianni F., de Martino L., Coppola R., de Feo V. (2013). Effect of essential oils on pathogenic bacteria. Pharmaceuticals.

[16] Fratianni F., Riccardi R., Spigno P., Ombra M.N., Cozzolino A., Tremonte P., Nazzaro F. (2016). Biochemical characterization and antimicrobial and antifungal activity of two endemic varieties of garlic (*Allium sativum* L.) of the campania region, southern Italy. J. Med. Food.

[17] Çobankara F.K., Altinöz H.C., Erganiş O., Kav K., Belli S. (2004). In vitro antibacterial activities of root-canal sealers by using two different methods. J. Endod..

[18] Mayrhofer S., Domig K.J., Mair C., Zitz U., Huys G., Kneifel W. (2008). Comparison of broth microdilution, Etest, and agar disk diffusion methods for antimicrobial susceptibility testing of *Lactobacillus acidophilus* group members. Appl. Environ. Microbiol..

[19] Saritha K., Rajesh A., Manjulatha K., Setty O.H., Yenugu S. (2015). Mechanism of antibacterial action of the alcoholic extracts of *Hemidesmus indicus* (L.) R. Br. ex Schult, *Leucas aspera* (Wild.), *Plumbago zeylanica* L., and *Tridax procumbens* (L.) R. Br. ex Schult. Front. Microbiol..

[20] Philip K., Malek S.N.A., Sani W., Shin S.K., Kumar S., Lai H.S., Serm L.G., Rahman S.N.S.A. (2009). Antimicrobial activity of some medicinal plants from Malaysia. Am. J. Appl. Sci..

[21] Salt T.A., Tocker J.E., Adler J.H. (1987). Dominance of Δ5-sterols in eight species of the Cactaceae. Phytochemistry.

[22] Ling W.H., Jones P.J.H. (1995). Minireview dietary phytosterols: A review of metabolism, benefits and side effects. Life Sci..

[23] Geran R.I., Greenberg N.H., Macdonald M.M., Schumacher A.M., Abbott B.J. (1972). Protocols for screening chemical agents and natural products against animal tumours and other biological systems. Cancer Chemother. Rep..

[24] Tan M.L., Sulaiman S.F., Najimuddin N., Samian M.R., Muhammad T.T. (2005). Methanolic extract of *Pereskia bleo* (Kunth) DC. (Cactaceae) induces apoptosis in breast carcinoma, T47-D cell line. J. Ethnopharmacol..

[25] Liew S.Y., Stanbridge E.J., Yusoff K., Shafee N. (2012). Hypoxia affects cellular responses to plant extracts. J. Ethnopharmacol..

[26] Pimenta F.C.F., Correia N.D.A., Albuquerque K.L.G.D., De Sousa D.P., Da Rosa M.R.D., Pimenta M.B.F., de Almeida R.N. (2012). Naturally occurring anxiolytic substances from aromatic plants of genus *Citrus*. J. Med. Plant Res..

[27] Kim J.M., Kim D.H., Park S.J., Park D.H., Jung S.Y., Kim H.J., Lee Y.S., Jin C., Ryu J.H. (2010). The *n*-butanolic extract of *Opuntia ficus*-*indica* var. *saboten* enhances long-term memory in the passive avoidance task in mice. Biol. Psychiatry.

[28] Abdul-Wahab I.R., Guilhon C.C., Fernandes P.D., Boylan F. (2012). Anti-nociceptive activity of *Pereskia bleo* Kunth. (Cactaceae) leaves extracts. J. Ethnopharmacol..

[29] Guilhon C.C., Abdul Wahab I.R., Boylan F., Fernandes P.D. (2015). Central antinociceptive and mechanism of action of *Pereskia bleo* Kunth. leaves crude extract, fractions, and isolated compounds. Evid.-Based Complement. Altern. Med..

[30] Elisabetsky E., Silva Brum L.F., Souza D.O. (1999). Anticonvulsant properties of linalool in glutamate-related seizure models. Phytomedicine.

[31] Davis M.I., Ronesi J., Lovinger D.M. (2003). A predominant role for inhibition of the adenylate cyclase/protein kinase A pathway in ERK activation by cannabinoid receptor 1 in N1E-115 neuroblastoma cells. J. Biol. Chem..

[32] Brand C.S., Hocker H.J, Gorfe A.A., Cavasotto C.N., Dessauer C.W. (2013). Isoform selectivity of adenylyl cyclase inhibitors: Characterization of known and novel compounds. J. Pharmacol. Exp. Ther..

[33] Council of Europe (2004). European Pharmacopeia.

[34] Jennings W., Shibamoto T. (1980). Qualitative Analysis of Flavour and Fragrance Volatiles by Glass Capillary Gas Chromatography.

[35] Davies N.W. (1990). Gas chromatographic retention indices of monoterpenes and sesquiterpenes on methyl silicone and Carbowax 20M phases. J. Chromatogr..

[36] Adams R.P. (2007). Identification of Essential Oil Components by Gas Chromatography/Mass Spectroscopy.

[37] Goodner K.L. (2008). Practical retention index models of OV-101, DB-1, DB-5, and DB-Wax for flavor and fragrance compounds. LWT-Food Sci. Technol..

[38] John Wiley & Sons (1998). Wiley Registry of Mass Spectral Data, with NIST Spectral Data CD Rom.

[39] Brand-Williams W., Cuvelier M.E., Berset C.L.W.T. (1995). Use of a free radical method to evaluate antioxidant activity. Food Sci. Technol..

[40] Singleton V.L., Rossi J.A. (1965). Colorimetry of total phenolics with phosphomolybdic-phosphotungstic acid reagents. Am. J. Enol. Vitic..

[41] Picerno P., Autore G., Marzocco S., Meloni M., Sanogo R., Aquino R.P. (2005). Anti-inflammatory activity of verminoside from *Kigelia africana* and evaluation of cutaneous irritation in cell cultures and reconstituted human epidermis. J. Nat. Prod..

[42] Petrella A., Ercolino S.F., Festa M., Gentilella A., Tosco A., Conzen S.D., Parente L. (2006). Dexamethasone inhibits TRAIL-induced apoptosis of thyroid cancer cells via Bcl-*_X_*_L_ induction. Eur. J. Cancer.

